# Identification of Gene-Specific Polymorphisms and Association with Capsaicin Pathway Metabolites in *Capsicum annuum L.* Collections

**DOI:** 10.1371/journal.pone.0086393

**Published:** 2014-01-27

**Authors:** Umesh K. Reddy, Aldo Almeida, Venkata L. Abburi, Suresh Babu Alaparthi, Desiree Unselt, Gerald Hankins, Minkyu Park, Doil Choi, Padma Nimmakayala

**Affiliations:** 1 Gus R. Douglass Institute and Department of Biology, West Virginia State University, Institute, West Virginia, United States of America; 2 Department of Plant Science, Plant Genomics and Breeding Institute, College of Agriculture and Life Sciences, Seoul National University, Seoul, Republic of Korea; Agriculture and Agri-Food Canada, Canada

## Abstract

Pepper (*Capsicum annuum L.*) is an economically important crop with added nutritional value. Production of capsaicin is an important quantitative trait with high environmental variance, so the development of markers regulating capsaicinoid accumulation is important for pepper breeding programs. In this study, we performed association mapping at the gene level to identify single nucleotide polymorphisms (SNPs) associated with capsaicin pathway metabolites in a diverse *Capsicum annuum* collection during two seasons. The genes *Pun1*, *CCR, KAS* and *HCT* were sequenced and matched with the whole-genome sequence draft of pepper to identify SNP locations and for further characterization. The identified SNPs for each gene underwent candidate gene association mapping. Association mapping results revealed *Pun1* as a key regulator of major metabolites in the capsaicin pathway mainly affecting capsaicinoids and precursors for acyl moieties of capsaicinoids. Six different SNPs in the promoter sequence of *Pun1* were found associated with capsaicin in plants from both seasons. Our results support that *CCR* is an important control point for the flux of p-coumaric acid to specific biosynthesis pathways. *KAS* was found to regulate the major precursors for acyl moieties of capsaicinoids and may play a key role in capsaicinoid production. Candidate gene association mapping of *Pun1* suggested that the accumulation of capsaicinoids depends on the expression of *Pun1*, as revealed by the most important associated SNPs found in the promoter region of *Pun1*.

## Introduction

Pepper (*Capsicum annuum L.*) is a crop of major agricultural and economic importance. It is known for its pungency, rich flavor, and nutritional value. World production of pepper in 2011 was estimated to be 29,939,029 metric tons; the United States alone recorded the production of 1,018,490 metric tons [1]. Pepper contributes a range of beneficial metabolites, such as carotenoids, flavonoid glycosides and vitamins, to the human diet [2]. The most unique metabolites are the alkaloids denominated by capsaicinoids, which make peppers pungent and are produced mainly in the placenta of the fruits [3]. Capsaicinoids have been widely used in food and for pharmaceutical purposes [4]–[6]. The most important pharmaceutical role of capsaicin is in pain perception. The transient receptor potential of vanilloid type 1 receptor (TRPV1) is activated by capsaicin in mammalian nociceptor cells, triggering inflammation and pain responses [7], [8]. Prolonged exposure to capsaicin numbs the TRPV1 over time, for long-term pain relief.

The use of molecular markers can save time and money in breeding programs by detecting particular traits before costly phenotyping is performed. Thus, genetic markers able to detect pungency and/or capsaicinoid profiles during the seedling stage are valuable tools in pepper breeding. Mazourek *et al*. [9] proposed a model integrating the capsaicin biosynthesis pathway and mapped genes. The acyl moieties of capsaicinoids are derived from catabolism of amino acids with subsequent fatty acid elongation [10], [11]. In later studies, Aluru *et al*. [12] reported that the transcript level of the placental-specific β-ketoacyl carrier protein synthase I (*KAS1*) was positively associated with pungency. Abraham-Juarez *et al*. [13] silenced *KAS* by virus-induced gene silencing in *Capsicum chinense* and created plants with undetectable levels of mRNA and capsaicinoids, thus providing further evidence for the important role of this gene in altering pepper pungency. A crucial branching point in the capsaicin pathway is the metabolite p-coumaric acid, which is also important in synthesis of a wide variety of secondary metabolites such as lignins, flavonoids, hydroxycinnamic polyamides and pigments[14]. Cinnamoyl CoA reductase (CCR) reduces coumaroyl, feruoyl and sinapoyl-CoA esters to their respective cinnamaldehydes; therefore, CCR is considered important in lignin biosynthesis and is a major control point of phenylpropanoid metabolic flux. It may have a role in determining capsaicinoid levels [15].

Capsaicinoids are alkaloids generated from the condensation of vanillylamine derived from the phenylpropanoid pathway and a variable branched chain fatty acid. A major dominant locus that alters capsaicin was mapped to chromosome 2 of pepper and named the *C* locus [16]–[18]. Kim *et al*. [19] identified SB2-66, a cDNA clone from a suppression subtractive hybridization library constructed from pungent *C. chinense* and further characterized to be homologous with acyl transferase. Interestingly, SB2-66 was found to express only in the placenta of pungent peppers. Stewart *et al.* [20] genotyped a mapping population with SB2-66 and noted that its relevant restriction fragment-length polymorphisms (RFLPs) co-segregated exactly with the pungency trait and mapped close to the *C* locus. Subsequently, Stewart *et al*. [21] sequenced a full-length transcript as well as genomic DNA, along with a 1.8-kb promoter, and named the locus *Pun1*. *Pun1* encodes AT3, an acyl transferase from the BAHD acyl transferase superfamily. Allelic tests for *Pun1* identified a 2.5-kb deletion unique to *C. annuum*. Later, the loss of pungency in *C. chinense, Capsicum frutescens* and *Capsicum chacoense* was found to be caused by species-specific independent events [21], [22]. Hill *et al*. [23] genotyped 43 pepper accessions, 40 belonging to *C. annuum*, and discovered seven homologs of *Pun1* and reported the presence of three acyl transferases. Nevertheless, *Pun1* is the only known locus to have a qualitative effect on pungency in *C. annuum* complex. Han *et al*. [24] demonstrated that *Pun*1 functions in capsinoid synthesis. Yumnam *et al*. [25] reported 79 single nucleotide polymorphisms (SNPs) in *Pun*1 from sequences of 15 pepper accessions of landraces from India. To date, no association mapping has been performed to measure the effects of individual SNPs on the accumulation of capsaicinoids.

Capsaicin and dihydrocapsaicin are the major capsaicinoids, and they differ only in the saturation of their fatty acid chain. Capsaicin and dihydrocapsaicin make up approximately 90% (66% and 22%, respectively) of total capsaicinoids [26]. Various *Capsicum* species and accessions within the species accumulate capsaicinoids in different proportions [27], [28]. Iwai *et al*. [29] indicated that capsaicin does not interconvert to dihydrocapsaicin, and some capsaicinoids do not undergo changes during different growth stages, which suggests unique regulatory effects on the expression of various enzymes in the capsaicin metabolic pathway.

In this study, we aimed to sequence *Pun1*, *CCR*, *KAS*, and hydroxycinnamoyl transferase (*HCT*) genes and use the underlying polymorphisms for association mapping to identify markers responsible for variation in capsaicinoids and the other metabolites in the capsaicin pathway among a diverse *C. annuum* population.

## Materials and Methods

### Plant material

We investigated 94 accessions of *C. annuum* from various countries representing a wide geographical area of the world (Table S1). These selfed accessions were grown in three replications during the summers of 2011 and 2012 (seasons 1 and 2). Genomic DNA isolation involved use of the DNeasy plant mini kit (QIAGEN cat# 69104).

### Metabolite profiling

Detailed metabolite profiling involved gas chromatography coupled with mass spectrometry (GC/MS) performed at the University of Illinois. For metabolic profiling, dried polar extracts were derivatized with 80 µl methoxyamine hydrochloride (20 mg ml-1) for 60 min at 50°C, 80 µl MSTFA for 120 min at 70°C, then 2-hr incubation at room temperature. An amount of 10 µL of the internal standard (hentriacontanoic acid, 10 mg/mL) was added to each sample before derivatization. Samples were analyzed on a GC/MS system (Agilent Inc, Palo Alto, CA, USA) consisting of an Agilent 7890 gas chromatograph, an Agilent 5975 mass selective detector, and a HP 7683B autosampler. Gas chromatography involved an HP-5MS capillary column (60 m×0.25 mm I.D. and 0.25-µm film thickness) (Agilent Inc, Palo Alto, CA, USA). The inlet and MS interface temperatures were 2500°C, and the ion source temperature was adjusted to 2300°C. An aliquot of 1 µL was injected with the split ratio of 10∶1. The helium carrier gas was kept at a constant flow rate of 1.5 ml min-1. The temperature program was 5-min isothermal heating at 700°C, followed by an oven temperature increase of 50°C min^−1^ to 3100°C and a final 10 min at 3100°C. The mass spectrometer was operated in positive electron impact mode (EI) at 69.9 eV ionization energy in m/z 30–800 scan range. The spectra of all chromatogram peaks were compared with those in electron impact mass-spectrum libraries NIST08 (NIST, MD, USA), W8N08 (Palisade Corp., NY, USA), and a custom-built library. To allow comparison between samples, all data were normalized to the internal standards in each chromatogram. The spectra for all chromatogram peaks were evaluated by use of the programs HP Chemstation (Agilent, Palo Alto, CA, USA) and AMDIS (NIST, Gaithersburg, MD, USA). Metabolome concentrations are reported as “(analyte concentration relative to hentriacontanoic acid) per gram Wet Weight” (relative concentration) (i.e., as target-compound peak area divided by the internal standard [IS] peak area [IS concentration is the same in all samples]): Ni = Xi × X-1hentriacontanoic acid × g wet weight^−1^. Hentriacontanoic acid (C31H62O2) is a fatty acid that is usually absent in any real sample we had dealt with. Calibration curves could not be built for all identified metabolites because some are not commercially available as pure standards. Relative concentration (RC) is an accepted way to compare the same metabolite between different samples but does not allow for comparisons between different metabolites within a sample because of different MSD responses to various compounds.

Capsaicinoids were extracted by diluting 100 mg dried powder with 2 mL pure acetonitrile after thorough mixing on a vortex. The mixture was incubated at 50°C for 1 hr followed by 1-hr sonication before centrifugation at 10,000 rpm for 15 min. The supernatant was filtered through a Phenomenex 0.2-µm PTFE membrane filter (Torrance, CA, USA) before analysis. Capsaicin and dihydrocapsaicin were quantified by use of a Waters high-performance liquid chromatography (HPLC) system equipped with 1525 binary HPLC pump, 2707 autosampler and 2998 Photodiode array detector (Waters Corp., Milford, MA, USA). Acetonitrile with 2% acetic acid was used as mobile phase at a flow rate of 0.6 ml/min. Separation of capsaicinoids involved an X-Bridge C18 column (4.6×150 mm; 5 µm) coupled with a guard column (Waters Corp.). Capsaicin and dihydrocapsaicin were detected at 280 nm. Injection volume was set to 10 µL. Retention times for capsaicin and dihydrocapsaicin were 9.3 and 9.7 min, respectively. Stock solutions of capsaicin and dihydrocapsaicin (Sigma–Aldrich) were prepared in acetonitrile for a linear standard curve from 12.5 to 500 ppm. Metabolite concentrations were normalized by log2 transformation before further analysis.

### Primer design and amplification

Gene-specific primers were designed with sequences available in Genbank for *HCT* (Genbank: EU616565), *CCR* (Genbank: EU616555), *KAS* (Genbank: HQ229922) and *Pun1* (Genbank: AY819029). Primer pairs were designed to amplify overlapping fragments of ∼500 to 1000 bp that covered full template sequences by use of Primer 3 software [30]. Sequences and annealing temperatures of primers are in Table S2. PCR amplification was performed in a total reaction volume of 50 µl containing 40 ng of genomic DNA with 25 µl GoTaq colorless master mix (Promega, Madison, WI, USA), 10 pmol each of forward and reverse primers, and completed with nuclease free water. Thermocycling conditions were an initial denaturing step of 95°C for 5 min, followed by 45 cycles of 95°C for 30 sec, corresponding annealing temperature for 30 sec and 72°C for 1 min, with a final extension step of 72°C for 2 min. Amplification of fragments was confirmed by visualization in a 1% agarose gel pre-stained with ethidium bromide under UV light. The amplified products were purified by polyethylene glycol precipitation.

### Sequence analysis

Sequencing involved the BigDye terminator cycle sequencing kit v.3.1 (cat# 4337455, Life Technologies) and an ABI 3130x/Genetic analyzer sequencer. Sequence fragments were aligned by use of the software Sequencher 4.9 (Gene Codes Corp., MI, USA). Exons and introns for each gene were determined by aligning available cDNA sequences of *Pun1* (Genbank: GU300812), *KAS* (Genbank: AF085148) and *CCR* (Genbank: EU616555) to the obtained genomic sequence with the software Spidey [31]. Chromosomal assignment and position on the physical map of candidate genes were deduced from the Whole Genome Sequence draft for hot pepper (CM334) (kindly provided by Drs. Park and Choi of Seoul National University). Phylogenetic trees were constructed for the four candidate genes. First, sequences for each gene were aligned in Sequencher 4.9 and the alignment was exported to MEGA 5.2 [32] to construct neighbor-joining trees. The nucleotide diversity (π) and Tajima’s test for selection were calculated on the alignments by use of DNASP 5.0 [33]. Consensus sequences for the promoter sequence of *Pun1* and intron sequences of *CCR* and *KAS1* were searched in the PLACE database for identification of known cis-regulatory elements [34].

### Candidate gene association mapping

Linkage disequilibrium (LD) was estimated as the correlation between all pairs of SNPs in individual candidate genes by use of the SNP & Variation Suite (SVS) v7.7.6 (www.goldenhelix.com). Haplotype blocks were computed with the default settings for the Gabriel *et al*. [35] algorithm imbedded in SVS v7.7.6. Haplotype frequencies for each defined haplotype block in all three genes were calculated by the estimation maximization (EM) method [36], with a frequency threshold of 0.01. Only SNPs with a minimum minor allele frequency > 0.1 were considered for LD studies and candidate gene association mapping. To visualize LD throughout the gene, heat maps were produced on the basis of pair-wise correlation estimates of all marker pairs. The Q and K matrices were adapted from previously performed simple sequence repeat (SSR) analysis [37]. Q matrix was adapted from K-5 cluster of SSR data obtained by use of Structure v2.2. The Mixed Linear Model (MLM) of TASSEL v3.0 was used for association mapping for *Pun1*, *KAS*1 and *CCR*. HCT did not undergo association mapping because of minimum minor allele frequency < 0.1 for SNPs discovered in this gene. The SNP P-values obtained were not subjected to sequential Bonferroni correction [38] or FDR [39]. Considering the sample size and number of polymorphisms used in our study, corrections for population structure and kinship were sufficient for association tests.

### Principal component analysis

Numeric principal component analysis (PCA) of the metabolic profiles involved use of SVS v7.7.6. Before PCA, concentrations of metabolites directly or indirectly involved in the capsaicinoid pathway for 93 pepper accessions were normalized by log2 transformation. Accessions were categorized by their recorded pungency level from HPLC analysis. Analysis of accessions grouped by pungency involved plotting values of the first two eigen vectors of PCA with use of SVS v7.7.6.

## Results

### Metabolic diversity

**Figure 1:**
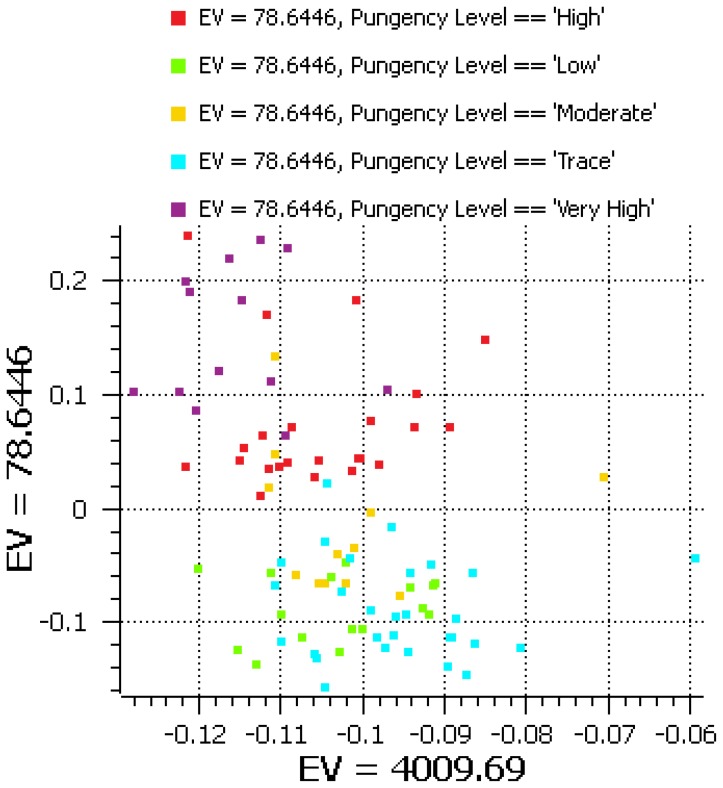
Principal component analysis of transformed concentrations of capsaicin pathway metabolites. Accessions are labeled by their *pun*gency level (EV  =  eigenvalue).

PCA with normalized concentration values for various metabolites (Table S3) obtained by GC/MS and HPLC revealed non-pungent peppers with trace amounts of capsaicin and those with low pungency and a few moderately pungent accessions remaining on the negative side of the Y-axis, with only moderate-, high- and very high-pungent accessions located on the positive side of the Y-axis (Fig. 1). Tepin produced the highest amount of capsaicin, followed by Prikkinu and Bird’s eye baby during season 1 (Table 1). In season 2, all peppers showed a significant decrease in capsaicin, which indicated a high degree of environmental variance. In season 2, Hot Ornamental Prairie Fire produced the most capsaicin, followed by Tepin and Bolivian rainbow.

**Table 1:** Top ten accessions for total capsaicinoid production.

Season 1		Season 2	
Log2 total capsaicinoids	Accession	Log2 total capsaicinoids	Accession
18.5616094	H114	4.660259827	CA05
18.51012019	H110	4.469676922	H114
17.37448703	H106	4.278490915	H102
17.04109434	H072	4.231831093	H138
16.79526245	H141	4.203551229	H077
16.2388068	H077	4.000636899	CA14
16.23223184	H138	3.920554658	H106
16.21818292	H102	3.806494182	H119
16.11034879	H067	3.765499939	H035
15.84204851	H105	3.757719495	CA17

### Association and diversity studies of *Pun1*

**Figure 2:**
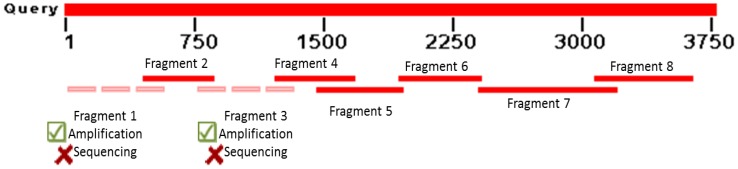
Schematic of fragments amplified and sequenced for *Pun1* with Genbank sequence used as a template.

All primer pairs belonging to the *Pun1* locus were successfully amplified in high-, moderate- and low-pungent accessions but not non-pungent peppers. This finding was expected because of a large deletion in the *Pun1* locus reported for non-pungent accessions. Because the fragments were purified for direct sequencing, the presence of homologous bands with similar size could not be resolved in 1% agarose gel nor sequenced, especially the amplicons of primer pairs *Pun*1_1 and *Pun*1_3 (Fig. 2). We obtained a fragment of 3197 bp for 43 genotypes, with the exception of a fragment that contained a 201-bp gap pertaining to the *Pun*1_3 fragment. Thus, only a 2996-bp portion of the gene was successfully sequenced from the available 3753-bp genomic sequence (Genbank: AY819029).

**Figure 3:**
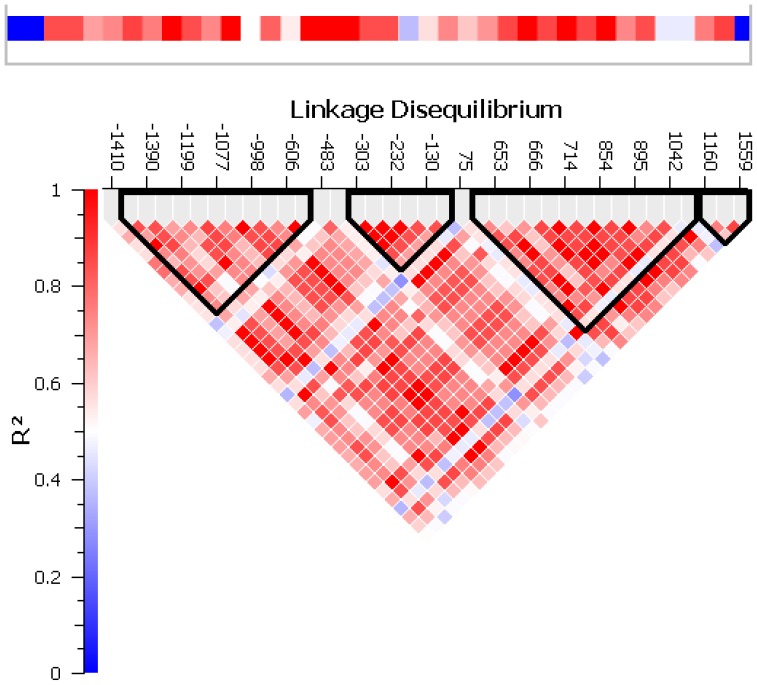
LD plot for *Pun1* showing defined haplotype blocks.

Alignment of exons from the cDNA sequence of *Pun1* to the *Capsicum* genome draft showed that *Pun1* is on the negative strand of chromosome 2 (Table 2). A total of 36 polymorphisms were identified in *Pun*1: 19 were localized in the promoter, seven in the first exon, seven in the intron and three in the second exon: 20 were transversions, 15 were transitions and one was an indel of two nucleotides (Table S4). SNP positions are numbered by orientation (down or upstream) and position from the transcription start site. Annotations of Cis regulatory elements for various SNP positions are presented in Table S4. SNPs –483 and –482 have the binding sequence of SEBFCONSSTPR10A, which is a silencing factor of resistance gene PR-10 in potato. In addition, SNP –116 has the sequence for MYB1LEPR which regulates defense related gene expression in tomato. The pattern of LD distribution in *Pun1* is presented in Fig. 3. On association mapping by the MLM approach, polymorphisms in *Pun1* were found associated with variation in six main metabolites in the capsaicin pathway (capsaicin, dihydrocapsaicin, isoleucine, leucine, pyruvate and valine). In addition, three more metabolites that are produced as result of deviation of the capsaicin pathway (naringenin, spermidine and vanillic acid) were found associated with the polymorphisms located in *Pun1*. The associated SNPs with respective p-values and correlation values are in Table S5. The six reported SNPs showed an association in both seasons. These six SNPs had the highest correlation values and allelic effects and are presented in Table 3. The SNPs causing synonymous and non-synonymous mutations are in Table 4: SNPs 653 and 654 were associated with capsaicinoids in season 1 and acyl moiety precursors; they cause non-synonymous mutations at 33 and 34 amino acids away from the active site, respectively.

**Table 2:** Chromosome positions of candidate genes on the *Capsicum* genome draft.

Gene region	Chromosome	Starting genome position	Ending genome position	Starting gene position	Ending gene position
Pun1					
Exon 1	chr02	120715906	120715133	1	772
Intron 1	chr02	120715132	120714794	773	1111
Exon 2	chr02	120714793	120714019	1112	1886
CCR					
Exon 1	chr03	233893926	233894046	1	121
Intron 1	chr03	233894047	233894333	122	408
Exon 2	chr03	233894334	233894489	409	564
Intron 2	chr03	233894490	233895067	565	1142
Exon 3	chr03	233895068	233895252	1143	1327
Intron 3	chr03	233895253	233895343	1328	1418
Exon 4	chr03	233895344	233895698	1419	1773
Intron 4	chr03	233895699	233896501	1774	2576
Exon 5	chr03	233896502	233896689	2577	2764
HCT					
Exon 1	chr07	44081214	44081621	1	408
Intron 1	chr07	44081622	44085264	409	4051
Exon 2	chr07	44085265	44086164	4052	4951

**Table 3:** Allele effects of *Pun1* single nucleotide polymorphisms (SNPs) associated with capsaicin in both growing seasons and dihydrocapsaicin in season 1.

SNP	Allele	Effect
Capsaicin season 1		
–1390	A	–2.586
	G	0
–1386	C	–2.62
	A	0
–1120	C	–2.365
	T	0
–1077	A	–2.39
	T	0
–130	C	–2.586
	T	0
–116	C	–2.62
	A	0
Capsaicin season 2		
–1390	A	–0.474
	G	0
–1386	C	–0.474
	A	0
–1120	C	–0.402
	T	0
–1077	A	–0.434
	T	0
–130	C	–0.474
	T	0
–116	C	–0.474
	A	0
Dihydrocapsaicin season 1		
–1390	A	–2.378
	G	0
–1386	C	–2.341
	A	0
–1120	C	–2.069
	T	0
–1077	A	–2.173
	T	0
–130	C	–2.378
	T	0
–116	C	–2.341
	A	0

**Table 4:** SNPs in the coding sequence of *Pun1.*

SNP	Exon	Type of mutation	Amino acid position	Original residue	Substituting residue
75	1	Non-synonymous	14	Aspargenine	Aspartate
302	1	Synonymous	89	Alanine	Alanine
653	1	Non-synonymous	206	Leucine	Serine
654	1	Non-synonymous	207	Valine	Isoleucine
666	1	Non-synonymous	211	Glutamine	Lysine
683	1	Synonymous	216	Leucine	Leucine
714	1	Non-synonymous	227	Glutamine	Glutamate
1160	2	Synonymous	259	Alanine	Alanine
1482	2	Non-synonymous	367	Lysine	Glutamate
1559	2	Synonymous	392	Argenine	Argenine

Four haplotype blocks were defined in *Pun1* (Fig. 3). Markers contained in each block and haplotype frequencies calculated with the EM method are in Table S6. Block 3 was the largest and contained 13 markers with six distinguished haplotypes. The estimated probabilities of only two haplotypes totaled 0.85 of the total probabilities for this block, whereas the remaining ∼0.15 is represented by four less-probable haplotypes. Block 4 was the smallest haplotype block, comprising the last three markers of the second exon in *Pun1*. For the remainder of the haplotype blocks, the same trend was observed, with most of the haplotype estimated probabilities (>0.80) represented by two haplotypes. The SNPs associated with capsaicin in both seasons were located in blocks 1 and 2. Block 1 contained the associated SNPs –1392, –1390, –1386, –1120 and –1077. Seven different haplotypes were estimated for this block.

**Figure 4:**
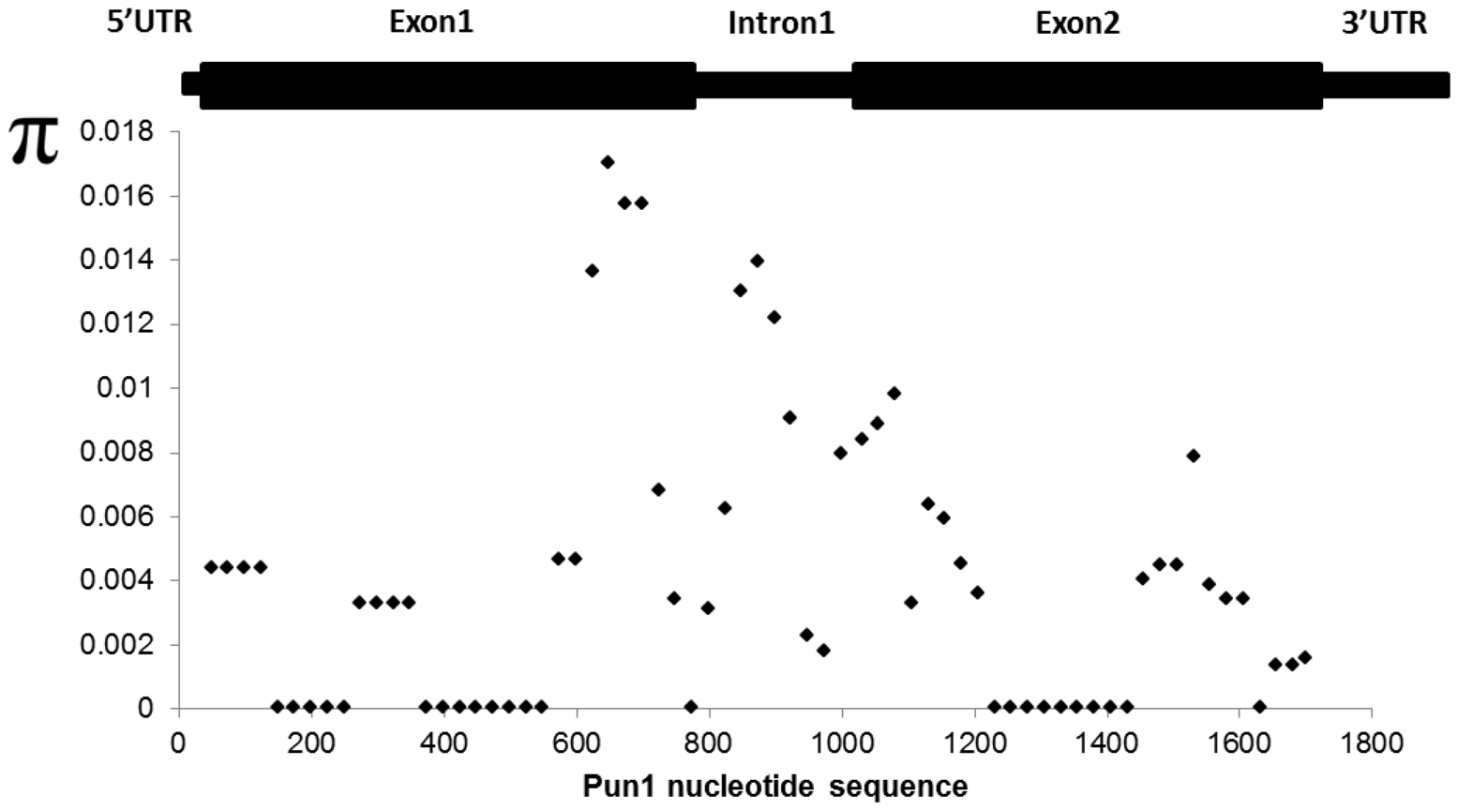
Nucleotide diversity (π) along the *Pun1* transcribed gene sequence.

The overall nucleotide diversity (π) for the *Pun1* locus including the promoter sequence was 0.0041 and that of the transcribed sequence 0.00387. When using a sliding window of 100 bp under a step size of 25 bp, the region near the active site (541–555 bp) had a nucleotide diversity of π  = 0, then after 575 bp π increased rapidly and peaked (π =  0.01699) in the region located near the end of the first exon at base 650 (Fig. 4). Subsequently, nucleotide diversity dropped near the splicing region of exon1 and increased again in the intron sequence (π =  0.0139), with a gradual drop to zero up to base 1432 located in exon 2. Tajima’s D for *Pun1* was calculated by genomic sequence alignment and only transcribed sequence alignment. Coding sequence alignment returned a D value of –0.665 considering 34 segregating sites. Meanwhile, genomic sequence alignment resulted in a lower D value of –1.027 calculated by data from 59 segregating sites, thus showing more evidence of purifying selection in *Pun1*. The discrepancy between the values can be explained by the number of segregating sites used, nevertheless, both analyses show that *Pun1* is under purifying selection as is common for domesticated traits.

Phylogenetic analysis was performed for the genomic and transcribed sequence alignments. The neighbor-joining algorithm separated all the accessions into two main clusters based on the polymorphisms located on the coding sequences of *Pun1* (Fig. S1). One clade was composed of only 9 accessions that included highly pungent Tepin. The second clade had two sister clades: one contained 28 accessions and the other the remaining eight accessions.

### Association and diversity studies of CCR

**Figure 5:**
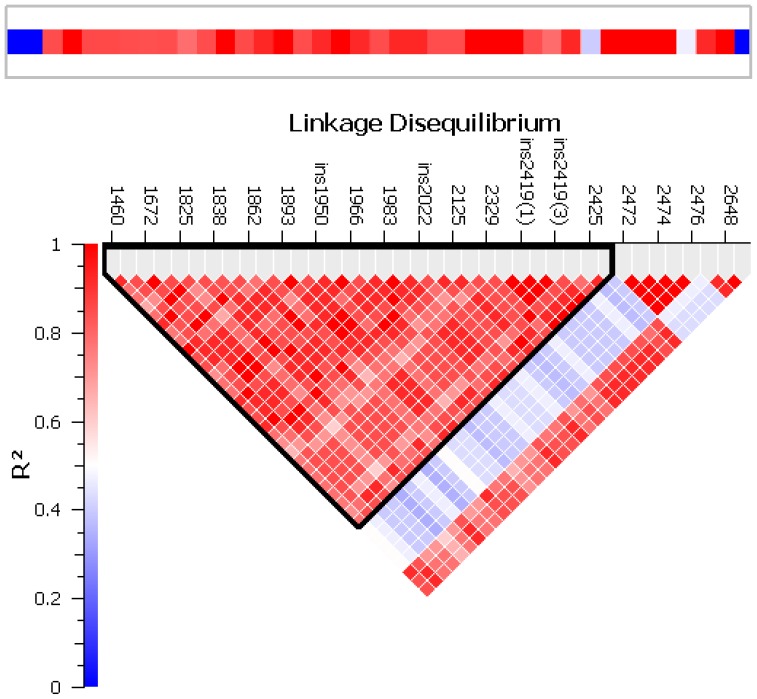
LD plot for *CCR* showing defined haplotype blocks.

CCR homologs are common in several plant families including *Capsicum*. The first primer pair of *CCR* (CCR_1) amplified two homologs, of ∼1.1 and 1.2 kb (data not shown). Primer pair CCR_2 yielded a single 1292-bp band and could be amplified across 53 pepper accessions. Alignment to the *Capsicum* genome draft positioned CCR on the (+) strand of chromosome 3 (Table 2). The full length of *CCR* in the genome is of 2764 bp; the alignment shows that *CCR* has 5 exons and 4 introns. The 1292-bp sequence extends from the beginning of the fourth exon at position 1419 to 2711 bp, toward the end of the gene. Sequence analysis showed that the NWYCY active site of CCR is well conserved in all accessions and is located in exon 4. Additionally, we report for the first time the presence of an intron between exons 4 and 5. A total of 32 polymorphisms were found in the *CCR* genomic fragment (Table S4). In all, 26 polymorphisms were located in the fourth intron, three in exon 4 and the remaining three in exon 5. Fifteen SNPs were transitions, and 13 were transversions. Additionally, we found two single-nucleotide insertions, another insertion with three nucleotides and a deletion of five nucleotides. Association mapping with MLM revealed *CCR* associated with caffeic acid and p-coumaric acid during season 1 (Table S5). A total of 14 polymorphisms were found associated with caffeic acid and also showed significant association with pyruvate, vanillate and p-coumaric acid. Haplotype analysis reported one block in *CCR* (Fig. 5). The block contained 28 markers, from the first polymorphism at 1460 bp to the SNP at 2426 bp. The first haplotype is represented by the major alleles and was estimated to have a probability of 0.52, while the rare alleles represented the second most frequent haplotype with an estimated probability of 0.30 (Table S6). Construction of a neighbor-joining tree allowed us to distinguish two main clades resolved by the polymorphisms located in *CCR* (Fig. S1). The largest clade contained 32 genotypes and the second contained the remaining 21 accessions.

**Figure 6:**
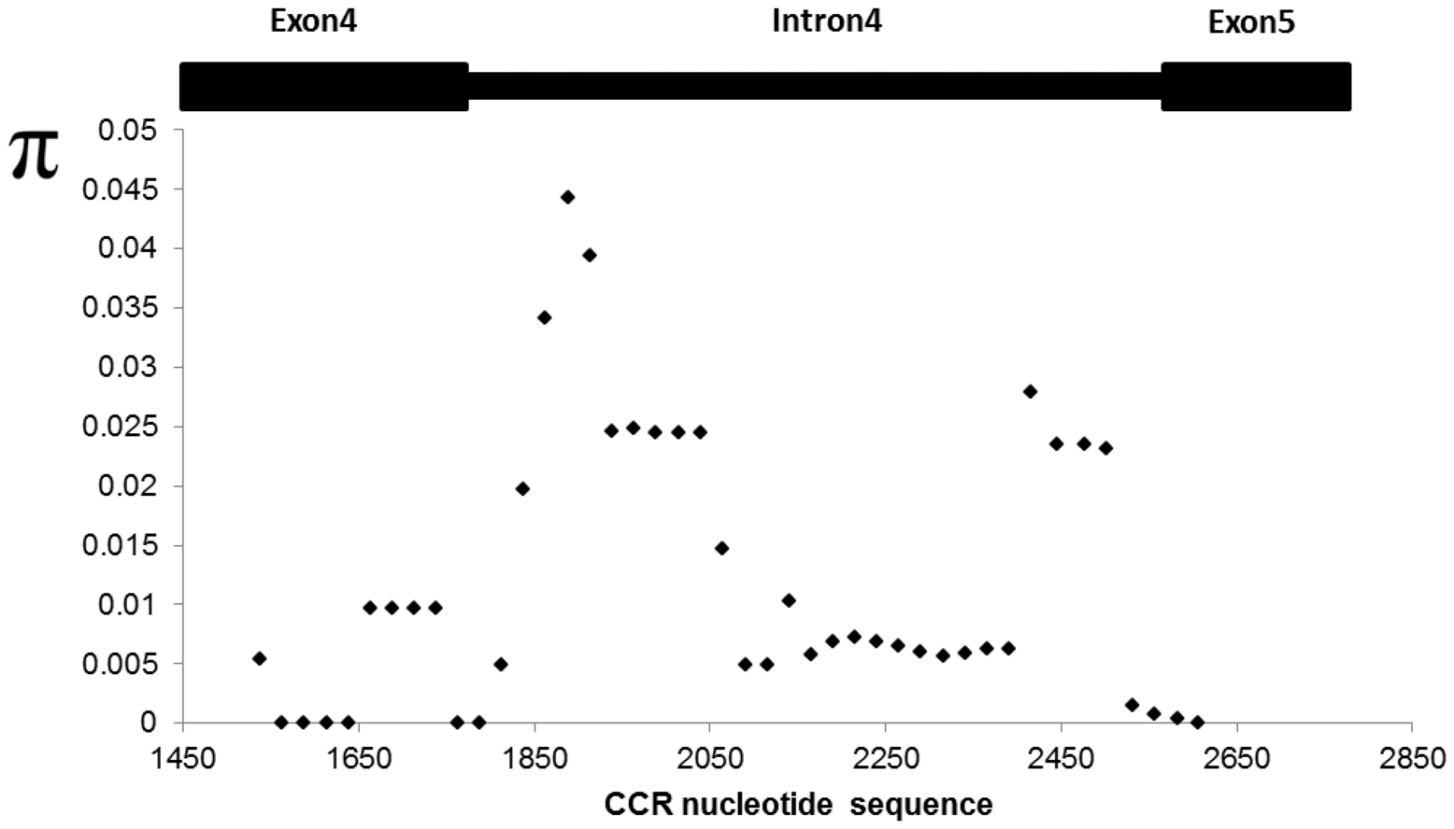
Nucleotide diversity (π) along the *CCR* gene sequence.

The overall nucleotide diversity (π) for *CCR* was 0.0011. With use of a sliding window of 100 bp under a step size of 25 bp, the highest nucleotide substitution was at about bp 1888 (π =  0.0443) located in intron 4, and the value was ∼2 times higher than the highest value observed for *Pun*1. The nucleotide diversity decreased to 0.0248 from bases 1938 to 2039, where the conserved motif for splicing factor is located (Fig. 6). Subsequently, nucleotide diversity dropped close to 0 near the splicing region of exon 5. Testing for selection revealed that *CCR* was under positive selection, with Tajima D  =  0.91, calculated with 47 segregating sites from 53 genotypes.

### Association and diversity studies of KAS

The sequence used for the study of *KAS* gene was the genomic isolate (Genbank: HQ229922), which was derived from the cDNA sequence of *C. chinense* (Genbank: AF085148). A BLASTX search of this sequence revealed *KAS*I and *KAS*II domains, and a nucleotide BLAST search aligned the sequence to the *KAS1* gene of tomato; hereafter, we refer to the gene studied as *KAS1*. Of eight primer pairs designed for *KAS1*, three were sequenced. We obtained a sequence of 1313 bases starting at position 149 of the *KAS1* gene and ended at base 1,461 from 62 genotypes by using the overlapping primer pairs for KAS1_1, KAS1_2 and KAS1_3. Alignment of the available cDNA sequence to the genomic sequence in Spidey revealed eight exons for this gene. The sequence obtained was extended from the last seven bases for the first exon to 232 bases for the second exon, while passing through an intron. No polymorphisms were detected in the coding regions, but six SNPs were identified in the intron. Association mapping with MLM revealed linkage of SNP 447 with isoleucine, leucine, pyruvate and valine, the major precursors of the fatty acid moieties in capsaicin (Table S5). The constructed neighbor-joining tree showed that Nepalese (H137) pepper has a distinct *KAS1* haplotype that separates it from the rest (Fig. S1). Nucleotide diversity for *KAS1* was calculated to be 0.0026 considering 29 segregating sites. Testing for neutrality indicated that *KAS1* is under negative selection, with Tajima D  =  –1.84.

### Association and diversity studies of HCT

For HCT, we amplified 778 bp in exon 2 using the primer pairs HCT_2 and HCT_3. The alignment did not reveal any SNPs with frequency > 0.1, so we did not perform association mapping. In fact, nucleotide diversity for HCT was 0.0003 and was calculated from seven segregating sites. The Tajima’s D was –2.044, indicating negative selection for the HCT locus.

## Discussion

Our association-mapping results revealed *Pun1* associated with six main metabolites in the capsaicin pathway (capsaicin, dihydrocapsaicin, isoleucine, leucine, pyruvate and valine) as well as three other metabolites produced from deviations of the capsaicin pathway (naringenin, spermidine and vanillic acid). Three SNPs, –483, –482 and 1559, controlled variation in major precursors for the acyl moieties pyruvate, valine, leucine and isoleucine, which are used in the synthesis of all known capsaicinoids. These metabolites are precursors of the fatty acid moieties that are used in the synthesis of capsaicinoids [9]. SNPs causing non-synonymous substitution of amino acids in the coding region affected only the levels of capsaicinoids and valine, leucine and pyruvate in season 1. *Pun1* greatly influenced the concentration of acyl moiety precursors, possibly because of the demand for production of the fatty acid moieties for capsaicinoid synthesis. Pyruvate is needed both for the synthesis of acetyl-CoA used in the fatty acid elongation pathway and as a precursor for the synthesis of valine, which is converted to iso-butyril and is elongated to the acyl moieties in two major capsaicinoids: capsaicin and dihydrocapsaicin [26]. Wahyuni *et al*. [2] studied metabolic profiles of *Capsicum spp* and found that variation in volatile compounds corresponded well to differences in pungency. In addition, our association mapping showed that *Pun1* is important in determining concentrations of narignenin, spermidine and vanillic acid, compounds resulting from deviation of the capsaicin pathway. Naringenin is a metabolite in the flavonoid pathway; 4-coumaroyl-CoA is derived from vanillin production and is converted to chalcone with subsequent isomerization to naringenin [40]. In fact, three SNPs (75, 653 and 714), associated with naringenin, were found in exon 1 of *AT3* and represented non-synonymous amino acid substitutions. Markers 653 and 714 were among the top three SNPs with the highest correlation values (r^2^  = 0.12) for naringenin. Three metabolites in the phenylpropanoid branch of the capsaicin pathway (coumaroyl-CoA, caffeoyl-CoA and feruoyl-CoA) are transferred to spermidine for the synthesis of hydroxycinnamic acid amides [41]. However, vanillic acid results from vanillin oxidation [42]. Our results showed that *Pun1* is a key regulator of the major metabolites in the capsaicin pathway.

For *CCR* in *Capsicum*, we could sequence only a fragment of the 1292 bp because of multiple bands. Other studies have described multiple *CCR* homologs for *Arabidopsis* and *Populus*, and in *Oryza* up to 26 *CCR* and *CCR*-like genes have been reported [43]. Similar to these studies, the first 400 bp of the *CCR* cDNA sequence aligned with another region of the pepper chromosome away from where the functional copy of *CCR* is located. This finding indicates the presence of the whole *CCR* gene family in *Capsicum*. Previous work on *CCR* has involved cDNA. In contrast, we used genomic DNA because intronic and genomic areas reveal more detailed information than the exons [44]. In this study, we reported data for the sequence of the fourth intron of the *CCR* gene in *C. annuum*. Additionally, our sequence analysis of *CCR* revealed that the conserved catalytic motif NWYCY of *CCR* [45] is located after two bases from the beginning of the fourth exon in *C. annuum*. As expected, *CCR* showed a major association with p-coumaric acid and caffeic acid. *CCR* is known to act on coumaroyl, caffeoyl and feruoyl-CoA, converting them to their respective aldehydes [46]. *CCR* activity is considered the first committed step in lignin biosynthesis [15], and our data support that the flux of coumarate and caffeate is highly controlled by *CCR*. Surprisingly, pyruvate and malonate were highly associated with *CCR* as well. Malonyl-CoA is used for fatty acid elongation and is synthesized from acetyl-CoA, which can be produced from pyruvate [9]. *CCR* appears to have an indirect influence in the fatty acid branch of the capsaicin pathway by determining the flow of p-coumaric acid used for capsaicin synthesis. However, three malonyl-CoA molecules are needed to synthesize chalcone from p-coumaric acid for flavonoid biosynthesis [40]. The association of *CCR* with malonate and pyruvate could be explained by *CCR* being the principal regulator of coumaroyl-CoA flux. In brief, *CCR* is a major determinant of p-coumaric acid, caffeic acid, pyruvate and vanillic acid concentrations in *Capsicum* fruits but also controls, in a minor manner, other metabolites in the capsaicin pathway.

Sequencing of *KAS1* in the current study was hampered by the presence of similar band-sized homologs. In accordance, Mazourek *et al*. [9] mapped the *KAS1* gene to seven different chromosomal locations in an integrated AFLP and RFLP map. Nevertheless, achieving direct sequencing of the fragments in the first intron indicates that this intron is highly conserved in sequence as well as size for all *KAS1* homologs of *C. annuum*. Our 2012 study revealed association of capsaicin and *KAS*. In a study by Aluru *et al*. [12], *KAS* expression was positively correlated with pungency, and silencing of the *KAS* gene led to lower levels of capsaicinoids as well [13]. In our study, all major precursors of capsaicinoid acyl moieties were found to be associated to *KAS1*. *KAS* genes are known to greatly affect the fatty acid composition of plants. For example, overexpression of *KASIII* in tobacco, *Arabidopsis* and rapeseed increased levels of 16:0 fatty acids [47]. Leonard *et al*. [48] report that the introduction of a *Cuphea wrightii* KAS gene homologous to *KASII* transformed in *Arabidopsis* shifted fatty acid profiles towards short 8:0 and 10:0 chains. In addition, glutamine and γ-amino butyrate were among the metabolites associated with *KAS1*. Catabolism of amino acids for producing branched acyl moieties in capsaicinoids requires several transfers of amino groups by branched-chain-amino-acid aminotransferase (BCAT) [9]. Although glutamate is considered the amino donor/acceptor in these steps, glutamine or γ-amino butyrate could also participate in the BCAT amino transfer reactions. Furthermore, γ-amino butyrate is a product of glutamate degradation. The low nucleotide diversity reported for HCT and the negative selection reflected by a –2.044 Tajima D value indicated that this gene is a locus of major importance for the phenylpropanoid pathway and plant development in general.

## Conclusions

Our results show *Pun1* as a regulator of major compounds in the capsaicin pathway, mainly capsaicinoids and also precursors for acyl moieties of capsaicinoids in *C. annuum*. Six different SNPs lying in the promoter sequence of *Pun1* were found associated with capsaicin in plants from two different growing seasons by the candidate gene association-mapping approach. The results of candidate gene association mapping of *Pun1* indicated that even though *Pun1* is the only known qualitative trait for pungency, accumulation of capsaicinoids depends more on different genomic regions regulating the expression of the enzymes in the pathway. Indeed, the most important SNPs were found in the promoter region of *Pun1*. We report the presence of an intron sequence for *CCR* in *C. annuum*, and an SNP in a conserved intron motif involved in pre-mRNA splicing affects concentrations of caffeic acid and p-coumaric acid. Our results also support CCR as an important control point for the flux of p-coumaric acid to specific biosynthesis pathways. Consistent with previous reports, we found that *KAS* regulates the major precursors of acyl moieties of capsaicinoids and may play a key role in capsaicinoid production. Functional characterization of these SNPs will provide further details into their effects on capsaicinoid metabolism, thus elucidating the mechanism of capsaicinoid level control.

## Supporting Information

Figure S1 **Neighbor-joining trees constructed with (A) transcribed sequence alignment of** ***Pun*** **1; (B) sequence alignment of** ***Pun1*** **including promoter; (C) sequence alignment of** ***CCR*** **; and (D) sequence alignment of** ***KAS*** **.**
(JPG)

Table S1 **Names of accessions in the study.**
(XLSX)

Table S2 **Primer pairs for candidate genes used in the study.**
(DOCX)

Table S3 **Log2 values for metabolite concentrations.**
(XLSX)

Table S4 **Details of polymorphisms in** ***Pun1*** **and annotations.**
(XLSX)

Table S5 **Association mapping of** ***Pun1, CCR*** **and** ***KAS*** **.**
(XLSX)

Table S6 **Haplotype frequencies of** ***Pun1, CCR*** **and** ***KAS.***
(XLSX)
