# Supplementary figures and images for: Identification of Gene-Specific Polymorphisms and Association with Capsaicin Pathway Metabolites in Capsicum annuum L. Collections
Source: PLoS One. 2014 Jan 27;9(1):e86393. doi: 10.1371/journal.pone.0086393 (PMC3903536; doi:10.1371/journal.pone.0086393)

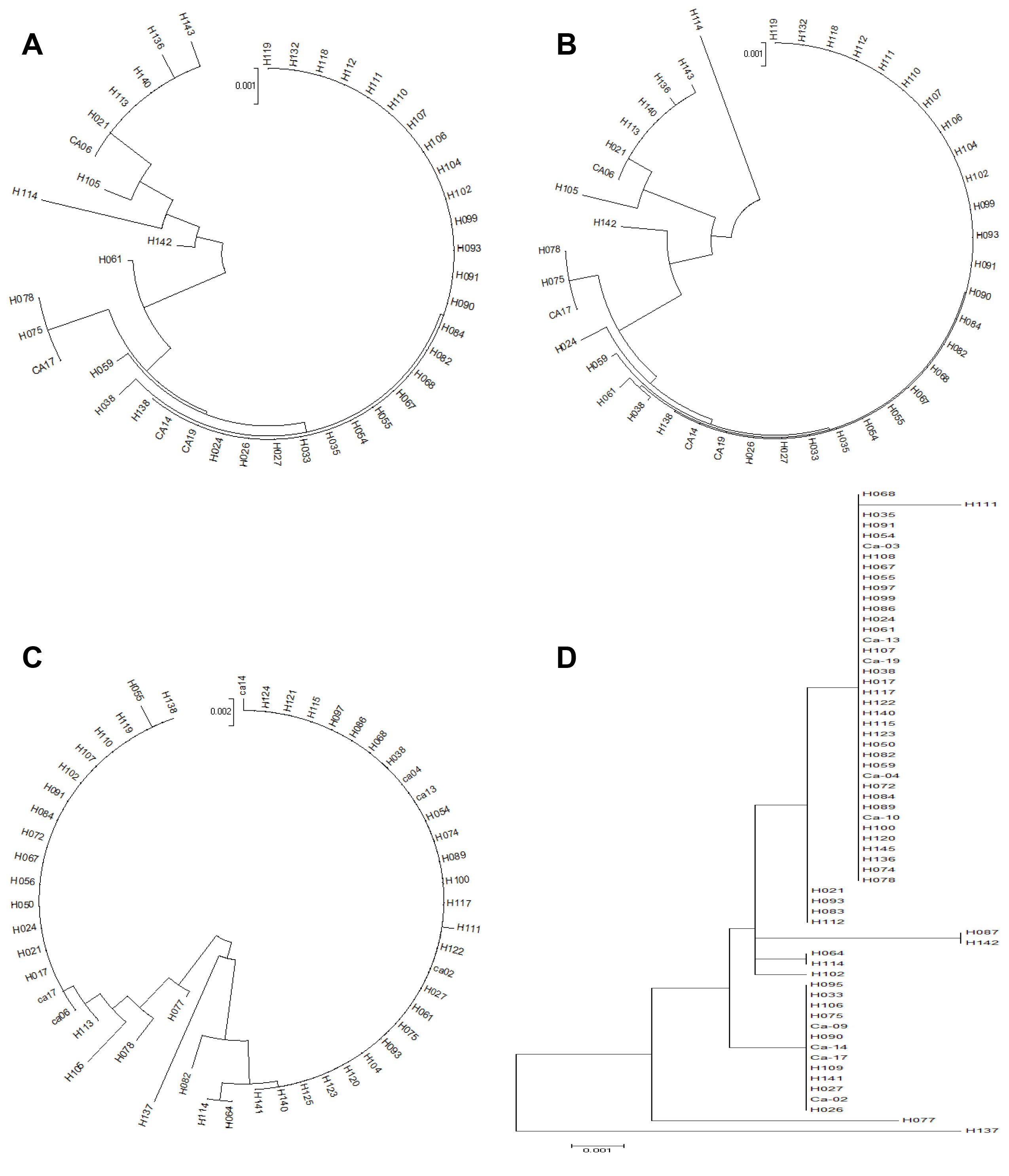

Supplement: Figure S1 — Neighbor-joining trees constructed with (A) transcribed sequence alignment of Pun 1; (B) sequence alignment of Pun1 including promoter; (C) sequence alignment of CCR ; and (D) sequence alignment of KAS . (JPG) [file pone.0086393.s001.jpg]
