# Supplementary material for: Identification of Gene-Specific Polymorphisms and Association with Capsaicin Pathway Metabolites in Capsicum annuum L. Collections
Source: PLoS One. 2014 Jan 27;9(1):e86393. doi: 10.1371/journal.pone.0086393 (PMC3903536; doi:10.1371/journal.pone.0086393)
Supplement: Table S2 — Primer pairs for candidate genes used in the study. (DOCX) [file pone.0086393.s003.docx]

Table S2. Primer pairs for candidate genes

| **Gene** | **Primer name** | **Annealing Temp. (°C)** | **Sequence** |
| --- | --- | --- | --- |
| *Pun*1 | *Pun*1_F1 | 55 | CCGCTCCACGAAAATGCACCT |
| *Pun*1 | *Pun*1_R1 | 55 | GGGGGTGGTGTTCATTTGATCATT |
| *Pun*1 | *Pun*1_F2 | 55 | CATCCCATTTTCAGTTCATAGACGACA |
| *Pun*1 | *Pun*1_R2 | 55 | AAGGGGTTGTTGCTGCTTTGC |
| *Pun*1 | *Pun*1_F3 | 55 | CGCTGTTCAAATCAAAATGCCAAA |
| *Pun*1 | *Pun*1_R3 | 55 | TGGCAAAGAAGGAACCCTCCA |
| *Pun*1 | *Pun*1_F4 | 55 | ATTGGTCCCTTGGCCGATGC |
| *Pun*1 | *Pun*1_R4 | 55 | CTCGTTGCACTGTGGCGTTG |
| *Pun*1 | *Pun*1_F5 | 55 | GGGATTGTCACGAAGGCGAGGT |
| *Pun*1 | *Pun*1_R5 | 55 | GGGGGTGAGAGAGGAAGGTTTGA |
| *Pun*1 | *Pun*1_F6 | 55 | GAAAATGTCAACCGGCCAGCA |
| *Pun*1 | *Pun*1_R6 | 55 | TCACCAATCTTGTGCGAAAAGCA |
| *Pun*1 | *Pun*1_F7-9 | 55 | TGCCTTGGGCGAATAATTGTGA |
| *Pun*1 | *Pun*1_R7-9 | 55 | CACGCCTTGCCCAGCTTTGT |
| *Pun*1 | *Pun*1_F10 | 55 | TGAGAGAAGGGAAACTGCCATTTGA |
| *Pun*1 | *Pun*1_R10 | 55 | TCCCTCTCTCTTCAATCAAACACCACA |
| KAS1 | KAS1_F1 | 52 | GAAGAAAGAATCAAGAATCAATGG |
| KAS1 | KAS1_R1 | 52 | GAGGGCCAGTGTACGAAGAT |
| KAS1 | KAS1_F2 | 52 | TGGAGAAAATGTGGGAATTTG |
| KAS1 | KAS1_R2 | 52 | AAGAAAAATGCAACATCAACACA |
| KAS1 | KAS1_F3 | 52 | GTTGTGGAATCAGCCAGTGA |
| KAS1 | KAS1_R3 | 52 | CCTTCAGAGGAGAAATCACGA |
| KAS1 | KAS1_F4 | 52 | CATAACTGGAATGGGCCTTG |
| KAS1 | KAS1_R4 | 52 | CCGTTCCACCTGCTACCATA |
| KAS1 | KAS1_F5 | 52 | GGTGCAAAGGGGATACAAGA |
| KAS1 | KAS1_R5 | 52 | ACCTCTTCTGGGGAAACTCC |
| KAS1 | KAS1_F6 | 52 | TTCCACACGAACAGGTGATG |
| KAS1 | KAS1_R6 | 52 | GGATCGGTTTTGTAATGCTGA |
| KAS1 | KAS1_F7 | 52 | CACAACCGTTATTTTGCTTACG |
| KAS1 | KAS1_R7 | 52 | CCGACGTTCACTTCATGTTG |
| KAS1 | KAS1_F8 | 52 | CGAATATTTGAATGGCAATGG |
| KAS1 | KAS1_R8 | 52 | TCAAGGTTTGTAGGGTGCAA |
| CCR | CCR_F1 | 52 | CGGAGGTTTCATTGCTTCTT |
| CCR | CCR_R1 | 52 | ATGTTTTGGCAGAGCCAGTT |
| CCR | CCR_F2 | 52 | CCACCGAGAAAGTTGTGGAC |
| CCR | CCR_R2 | 52 | TACTAATCACTCGAATAGATTCGTC |
| HCT | HCT_F1 | 52 | TGAGAGAATCGACGATGGTG |
| HCT | HCT_R1 | 52 | TACTCGATGTGGGGGAACTT |
| HCT | HCT_F2 | 52 | TTTAAATGTGGGGGAGTTTCC |
| HCT | HCT_R2 | 52 | AATTTTGCTGGCTGCATACC |
| HCT | HCT_F3 | 52 | CCTATGAGATGTTGGCAGGA |
| HCT | HCT_R3 | 52 | GCTTCATGTGTTCTGCTTGC |
